# The Regulation of MS-KIF18A Expression and Cross Talk with Estrogen Receptor

**DOI:** 10.1371/journal.pone.0006407

**Published:** 2009-07-28

**Authors:** Margalit Zusev, Dafna Benayahu

**Affiliations:** Department of Cell and Developmental Biology, Sackler School of Medicine, Tel-Aviv University, Tel-Aviv, Israel; University of Valencia, Spain

## Abstract

This study provides a novel view on the interactions between the MS-KIF18A, a kinesin protein, and estrogen receptor alpha (ERα) which were studied *in vivo* and *in vitro*. Additionally, the regulation of MS-KIF18A expression by estrogen was investigated at the gene and protein levels. An association between recombinant proteins; ERα and MS-KIF18A was demonstrated *in vitro* in a pull down assay. Such interactions were proven also for endogenous proteins in MBA-15 cells were detected prominently in the cytoplasm and are up-regulated by estrogen. Additionally, an association between these proteins and the transcription factor NF-κB was identified. MS-KIF18A mRNA expression was measured *in vivo* in relation to age and estrogen level in mice and rats models. A decrease in MS-KIF18A mRNA level was measured in old and in OVX-estrogen depleted rats as compared to young animals. The low MS-KIF18A mRNA expression in OVX rats was restored by estrogen treatment. We studied the regulation of MS-KIF18A transcription by estrogen using the luciferase reporter gene and chromatin immuno-percipitation (ChIP) assays. The luciferase reporter gene assay demonstrated an increase in MS-KIF18A promoter activity in response to 10^−8^ M estrogen and 10^−7^M ICI-182,780. Complimentary, the ChIP assay quantified the binding of ERα and pcJun to the MS-KIF18A promoter that was enhanced in cells treated by estrogen and ICI-182,780. In addition, cells treated by estrogen expressed higher levels of MS-KIF18A mRNA and protein and the protein turnover in MBA-15 cells was accelerated. Presented data demonstrated that ERα is a defined cargo of MS-KIF18A and added novel insight on the role of estrogen in regulation of MS-KIF18A expression both *in vivo* and *in vitro*.

## Introduction

Kinesins are microtubule-dependent motor proteins, with more than 45 members expressed in mammalian cells. Kinesins are classified based on three structural and functional regions: (i) a motor domain with microtubule binding site and a catalytic ATPase domain; (ii) central alpha-helical-coiled coil region which possesses protein-protein interactions and (iii) tail which interacts with cargo [1]. Different kinesin proteins share high homology of their motor domain but diverge considerably in the cargo-binding tail. Structural heterogeneity of kinesins is the basis for their diverse functions in various cellular processes including transport of cargoes such as membranous organelles, macromolecular complexes and mRNA [2]–[7]. It has been shown that kinesins play a role in trafficking directed towards the cell periphery, for example motility from the Golgi to the plasma membrane [8].

MS-KIF18A is a member of Kinesin-8 sub family [9] which was cloned from the marrow stromal cells and characterized by bioinformatic and biochemical means [10], [11]. Estrogen receptor alpha (ERα) was identified as a cargo for MS-KIF18A. We also suggested a role for this kinesin in estrogen signaling pathway [12]. Estrogen has pivotal functions in both female and male physiology and has been recognized as a regulator of bone remodeling in maintaining of bone mass and keeping the balance between bone formation and resorption [13]–[15]. Estrogen deficiency *in vivo* is recognized during post-menopause or following ovariectomy and associated with an increase of osteoclasto-genesis and decrease in osteogenesis that lead to bone destruction [16]–[19]. Estrogen hormone action affects cell proliferation and differentiation via the estrogen receptors (ERs). The ERs are expressed in various cells including osteoblasts [12], [20]–[24], osteocytes [25] osteoclasts [26] and mammary epithelial cells [27]. Specifically, ERα is identified in two isoforms: 66 kDa and 46 kDa, the shorter form lacking a ligand-independent activation function domain 1 (AF-1) [28], [29].

Steroid hormone binding to the receptors leads to a rapid (second – minutes) non-genomic signal transduction or to a prolonged genomic signaling [30]. The non-genomic pathway is mediated by activation of Mitogen Activated Protein Kinase (MAPK) proteins such as p38 and ERK1/2 [31] and increase in Ca^2+^ ion concentration [32], [33] or Inositol 1, 4, 5-trisphosphate (IP3) [34]. Such activation controls various cellular activities including cell proliferation, response to inflammation mediated via inhibition of NF-κB activation [35] and anti-apoptotic events [36]–[38]. The prolong estrogen action occurs within 30–60 minutes where the receptor is translocated to the nucleus and leads to genomic response. The ERα binds directly to estrogen response elements (EREs) [39] or indirectly via accessory proteins on AP-1 or Sp-1 binding sites [40] on promoters of target genes. The ERα translocation to the nucleus is a dynamic process regulated by ATP activity or by ligand-induced conformational changes and proteasome function. Depletion of ATP retards the intra-nuclear mobility of un-liganded ERα and causes the receptor redistribution to the cytoplasm [41]. When cells' treated with either 17βE_2_ or tamoxifen prior to ATP depletion the ERα was less mobile, more prominent in the nucleus and reduced the shuttling to the cytoplasm [42]. The ERα shuttling as ATP-dependent phenomena implies a role of motor protein in this process; however, thus far a candidate for such protein was not identified.

In this study, we presented two views on the MS-KIF18A - ERα cross talk: one aspect investigated the complex formation between MS-KIF18A and ERα and the second studied the regulation of MS-KIF18A expression under estrogen paradigm. The nature of interactions between ERα and MS-KIF18A was demonstrated using recombinant and endogenous proteins by immunoprecipitation (IP) and western blot (WB) assays. MS-KIF18A mRNA expression was analyzed *in vivo* in bone marrow cells or *in vitro* in a pre-osteogenic MBA-15 cells and breast carcinoma MCF-7 cells that are estrogen responsive cells. Estrogen effects on the binding of ERα and pcJun to MS-KIF18A promoter was studied by chromatin immunoprecipitation (ChIP) and the activation of the promoter was analyzed by luciferase reporter assay. The regulation of MS-KIF18A protein expression and turnover was explored by metabolic labeling and immunological analysis. The present research provides a novel view on regulation of MS-KIF18A and its' association with ERα and significantly contributes to the profound understanding of estrogen mediated activities.

## Results

**Figure 1:**
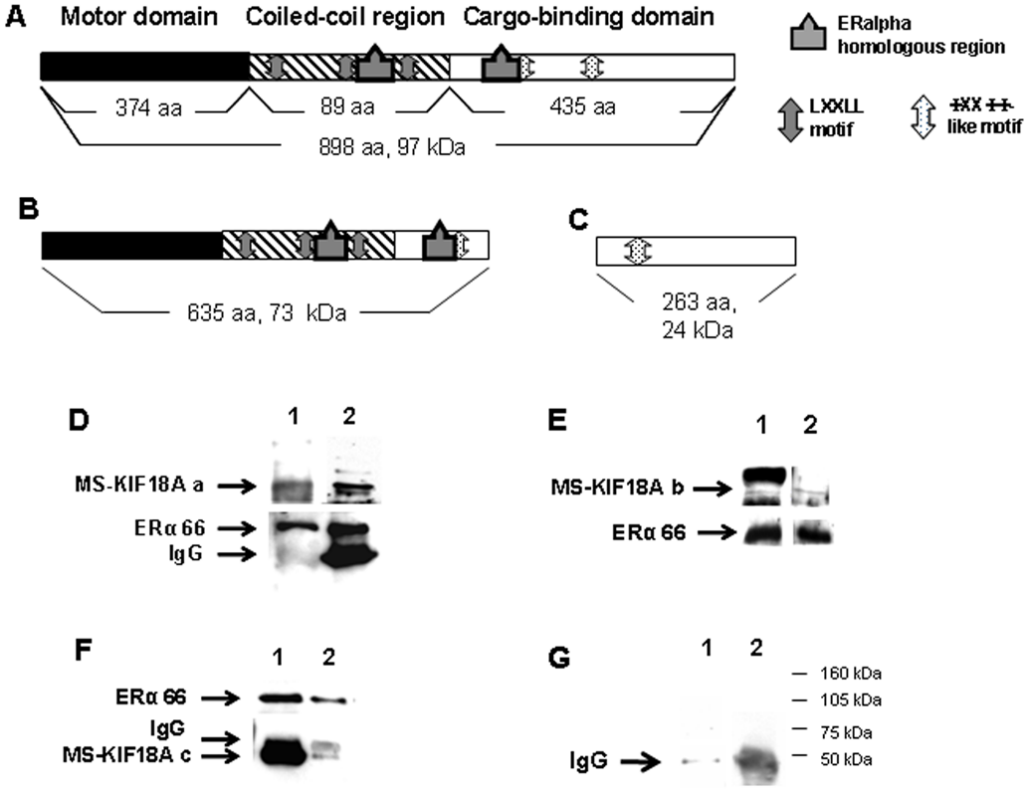
Association between MS-KIF18A and ERα recombinant proteins. Schematic illustration of full length MS-KIF18A (A); truncated MS-KIF18A construct 1–635 aa (B); truncated MS-KIF18A construct 635–898 aa (C). (D–F) Co-IP experiments of MS-KIF18A constucts with recombinant ERα and WB with monoclonal anti-MS-KIF18A (1) and anti-ERα (2). Full length MS-KIF18A (D), MS-KIF18A constucts 1–635 aa (E), MS-KIF18A constucts 635–898 (F). Pull down with beads only (1); IP with anti-MS-KIF18A and WB with anti-ERα (2) (G). The results are representative from the set of at least three independent experiences.

**Figure 2:**
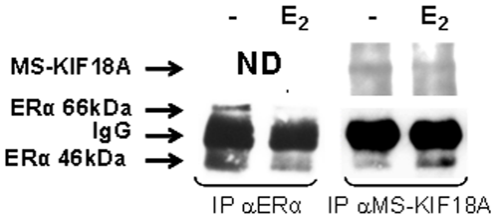
Estrogen-dependent association between MS-KIF18A and ERα. MBA-15 cell lysates were IPed with anti-ERα or anti-MS-KIF18A, and analyzed by WB. Results are of representative experiment of a series repeated five times.

The association between MS-KIF18A and a putative cargo; ERα was demonstrated in our laboratory in earlier study [12]. Currently, we elaborated on the interactions between these proteins using an *in vitro* pull down assay which applied recombinant proteins. We used three recombinant isoforms of MS-KIF18A: full length of MS-KIF18A (Figure 1A) and truncated forms: 1–635 AA that includes the motor domain and the coiled-coil region (Figure 1B) and 635–898 AA that contains the cargo binding domain (Figure 1C). The protein association between the three MS-KIF18A constructs and ERα was demonstrated by Co-IP and WB using three antibodies: a polyclonal anti-MS-KIF18A which identifies an epitope at the motor domain, a monoclonal anti-MS-KIF18A which identifies an epitope at the cargo-binding domain and anti-ERα (Figure 1D–1F). In addition, we previously demonstrated interactions of endogenous proteins in MBA-15 cells and revealed an association of MS-KIF18A with both 46 kDa and 66 kDa ERα isoforms [12]. Herein, we analyzed the 17βE_2_ effect on this complex formation in MBA-15 cells. Cells were pre-incubated in steroid-free serum for 48 h and then challenged with 10^−8^ M 17βE_2_ for 16 h followed with IP using either anti-MS-KIF18A or anti-ERα and analyzed by WB. In the treated cells, we detected a reduction in ERα appearance and an increase in MS-KIF18A-46 kDa ERα complex formation (Figure 2).

**Figure 3:**
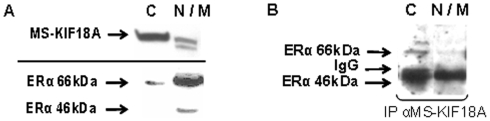
Sub-cellular distribution of ERα and MS-KIF18A in cells fractionated to cytoplasm (C) and nuclear/membrane (N/M) compartments. Whole lysates (A) and IP (B) of fractionated cells were analyzed with anti-MS-KIF18A and anti-ERα. Results demonstrate a representative experiment of four independent repeats.

**Figure 4:**
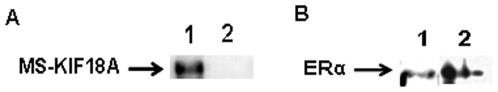
Interaction between NF-κB and MS-KIF18A or ERα. IP with anti-p50 (1) with anti-p65 (2) WB performed with anti-MS-KIF18A (A) with anti-ERα (B). Results revealed an association between MS-KIF18A and p50, but not with p65 while ERα interacts with both forms of NF-κB; p65 and p50. Results demonstrate a representative experiment of three independent repeats.

MS-KIF18A sub-cellular distribution and co-expression with ERα was analyzed at the cytoplasm (C) and nuclear/membrane (N/M) compartments. The ERα expression was identified at higher level in the nucleus, while MS-KIF18A was prominently localized in the cytoplasm (Figure 3A). IP with anti-MS-KIF18A and WB analysis with anti-ERα detected the MS-KIF18A-ERα complex mainly at the cytoplasm (Figure 3B). To elaborate on the role of MS-KIF18A in ERα signaling pathway we analyzed the interactions of ERα and MS-KIF18A with NF-κB (p65 and p50 subunits). Cell lysates were immunoprecipitied with antibodies towards p65 and p50 subunites of NF-κB and follwed with WB analysis using anti-MS-KIF18A. An association was found between MS-KIF18A and p50, whereas no interactions with p65 was observed (Figure 4A). However, protein complex was noted between ERα and both forms of NF-κB (Figure 4B).

**Figure 5:**
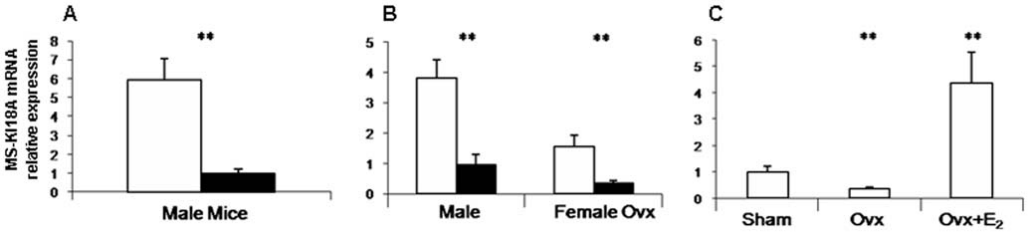
MS-KIF18A mRNA expression *in vivo*. Total RNA from bone marrow cells were harvested from mice (A) and rats (B, C) and analyzed by qRT-PCR. (A) mRNA expression in bone marrow cells derived from young 4 month (white bars) and old 12 month (black bars) male mice; (B) Young 3 month (white bars) and 14 month old (black bars) male and OVX female rats; (C) Sham, OVX and OVX+E_2_ female rats. MS-KIF18A mRNA expression was normalized to G3PDH expression levels. Results are presented as mean values +/− SD obtained from triplicates for each data point.

The role of estrogen on MS-KIF18A mRNA expression was analyzed *in vivo* and in cell culture. MS-KIF18A mRNA measured *in vivo* on RNA isolated from bone marrow cells harvested from rats and mice and correlated the expression levels in relation to animal age (Figure 5A, 5B). Studying mice, we measured 6-folds higher mRNA levels in young males then in old animals (p = 0.0019, Figure 5A). In rats, the expression of the mRNA was 4-folds higher in young male animals than in old ones (p = 0.0022, Figure 5B). In young female OVX-rats the level of mRNA was 3-folds higher compared to old ones (p = 0.0047, Figure 5B). All together, we noted a higher MS-KIF18A mRNA levels in bone marrow of young animals that was decreased with age. In addition, we analyzed sham rats that expressed MS-KIF18A mRNA levels 3-folds higher as compared to OVX-rats (p = 0.0084, Figure 5C). When OVX-rats were treated with 17βE_2_, a 12.5-folds increase in mRNA levels was detected (p = 0.0078, Figure 5C). These results provide the *in vivo* evidence of estrogen impact on the regulation of MS-KIF18A expression.

**Figure 6:**
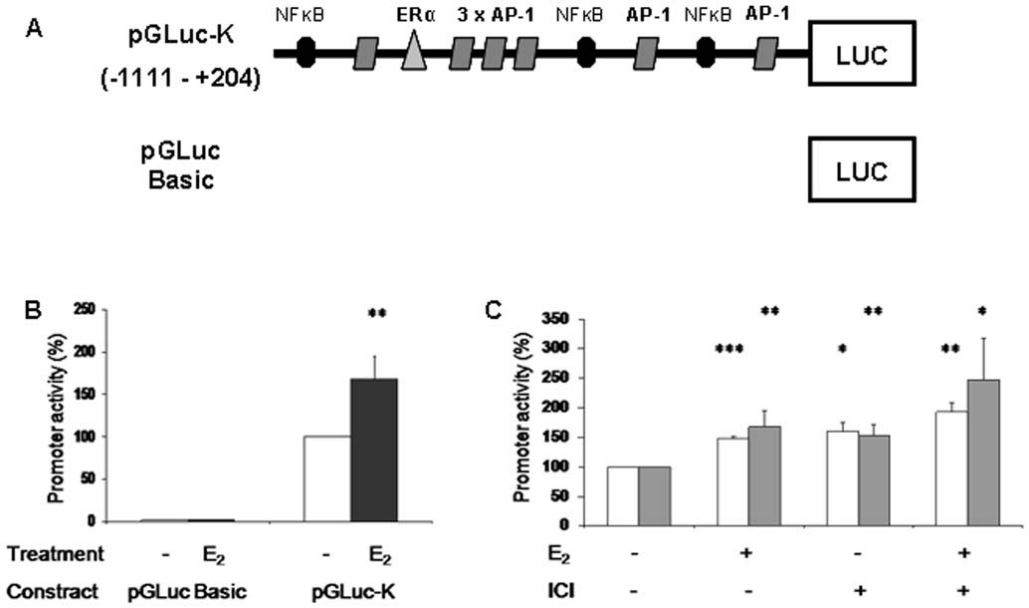
Luciferase measurements of MS-KIF18A promoter activity. (A) Schematic illustration of MS-KIF18A promoter-luciferase reporter constructs. MCF-7 cells transfection with MS-KIF18A promoter cloned in luciferase reporter plasmid (pGLuc-K) or promoter less pGL3-basic along with β-galactosidase vector. (B) Cells treated (black bars) or not (white bars) with 17βE_2_ (10^−8^ M) for 24 h; (C) 17βE_2_ (10^−8^ M) or/and ICI-182,780 (10^−7^ M) were added to the cultures for 1 h (white bars) or 24 h (gray bars). Promoter activities are expressed as luciferase values normalized for β-galactosidase levels. A value of 100% was given to the basal promoter activity elicited by the pGLuc-K construct in the absence of any treatment. Results are mean±SD of 3 independent experiments, performed in duplicates.

The stimulatory effect of 17βE_2_ on MS-KIF18A mRNA expression led us to analyze the transcription regulation using a reporter gene assay. We analyzed in silico, the 1500 bp upstream to the TSS of the MS-KIF18A gene. This region was predicted as putative promoter and mapped for transcription factors (TFs) binding sites and regulatory elements. Bioinformatics analysis using MatInspector software enables to identify the promoter region and the TF binding sites and regulatory elements. Specifically, we mapped a non-palindrome half-site ERE that binds ER directly and AP-1 site that binds ER via accessory proteins such the pcJun. We amplified this genomic region by PCR and cloned into pGLuc vector upstream to luciferase reporter gene (pGluc-K) (Figure 6A). The cloned pGluc-K plasmid was co-transfected with β-galactosidase (β-GAL) plasmid in MCF-7 cells that were treated with 10^−8^ M 17βE_2_ or/and 10^−7^ M ICI 182,780 for 1 h or 24 h. The promoter activity was quantified by luciferase activity normalized to β-GAL (which indicates the transfection efficiency). In 17βE_2_ treated cells' we measured an increase in luciferase activity: 1.5-folds after 1 h (p = 0.0001) and 1.7-folds after 24 h (p = 0.01) as compared to control cells. Cells treated with ICI-182,780 demonstrated an increase of luciferase activity; 1.6-folds following 1 h (p = 0.02) and 1.5-folds after 24 h (p = 0.006) as compared to untreated cells. The combined treatment of 17βE_2_/ICI-182,780 resulted with a similar increase of luciferase activity after 1 h (p = 0.0012) and increase 2.5-folds after 24 h (p = 0.023) of treatment (Figure 6C). No activity in transfected cells with pGLuc-basic plasmid was detected (Figure 6B).

**Figure 7:**
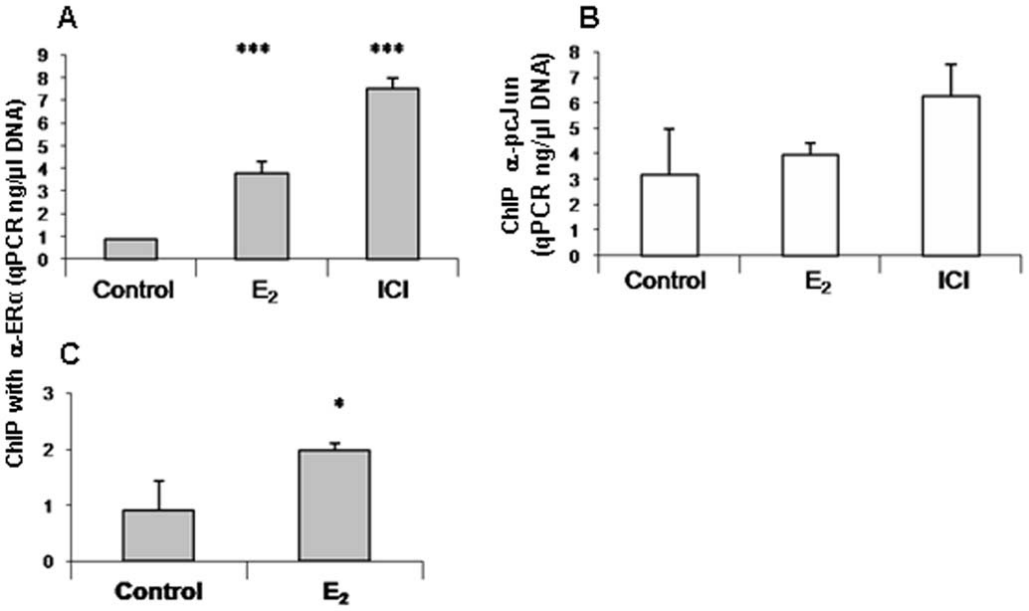
Chip assay of ERα and AP-1 binding to MS-KIF18A promoter. A-C Bar histogram of qPCR analysis of amplified MS-KIF18A promoter in MCF-7 ChIPed by anti-ERα (A) or by anti-pcJun (B), and in MBA-15 cells were ChIPed by anti-ERα (C). All the results presented as mean values +/− SD obtained from three different experiments each performed in triplicates for each data point.

ChIP assay was applied to correlate the endogenous regulation of promoter activity. We used antibodies to ERα and pcJun to measure their binding to MS-KIF18A promoter in MCF-7 and MBA-15 cells (Figure 7). In MCF-7 cells treated with 17βE_2_ for 60 min, we noted 4-folds increase in ERα binding (p = 0.0007, Figure 7A) while no change in the level of pcJun binding (Figure 7B) was observed. When the MCF-7 cells were treated with ICI-182,780, binding of ERα was 9-folds higher (p = 0.0001, Figure 7A) and the binding of pcJun was 2-folds higher (Figure 7B) as compared to untreated cells. Treatment of MBA-15 cells with 17βE_2_ for 2 h then ChIPed with anti-ERα revealed a 2-folds increase of ERα binding to the analyzed promoter (p = 0.0281, Figure 7C) as compared to untreated cells.

**Figure 8:**
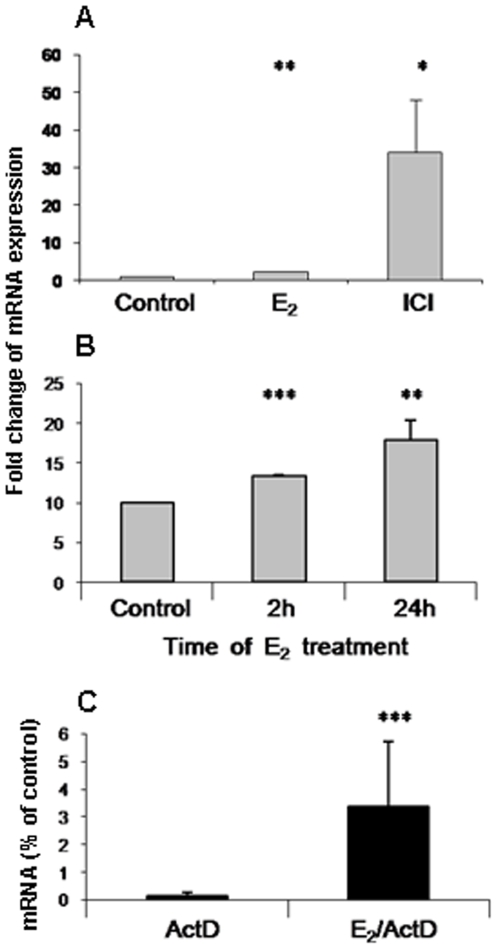
MS-KIF18A mRNA expression presented by bar histogram of qPCR analysis from (A) MCF-7 cells treated or untreated for 60 min with 10^−8^ M 17βE_2_ (E_2_) or 10^−7^ M ICI-182,780 (ICI) (B) MBA-15 cells treated with 17βE_2_ for 2 h and 24 h. (C) Effect of Act D on the stability of MS-KIF18A mRNA in MBA-15 cells treated in presence of 17βE_2_ for 6 h. The MS-KIF18A mRNA is samples were normalized to G3PDH for each data point. Results are presented as mean values +/− SD obtained from three different experiments each performed in triplicates for each data point.

The consequence of 17βE_2_ or ICI-182,780 regulation of ERα and pcJun binding to MS-KIF18A promoter and its' activation led us to study the MS-KIF18A mRNA expression level under this paradigm. The message expression level was quantified by comparative qRT-PCR (Figure 8). MCF-7 cells response to 17βE_2_ treatment for 1 h measured 2-folds increase (p = 0.0014) while treatment with ICI-182,780 induced 34-folds increase (p = 0.014) of MS-KIF18A mRNA expression (Figure 8A). MBA-15 cells treatment with 17βE_2_ resulted with 1.3-folds elevation mRNA levels after 2 h (p = 0.0001) and 1.8- folds after 24 h (p = 0.0053) as compared to untreated control (Figure 8B).

Actinomycin D (ActD) is an inhibitor of RNA synthesis. We measured the mRNA levels in cells treated by this drug in presence or absence of 17βE_2_ and noted a 25-folds increase in mRNA levels in ActD/E_2_ treated cells as compared to cells' treated by ActD only (p = 0.0001, Figure 8C) indicating mRNA stabilization in cells treated with estrogen.

**Figure 9:**
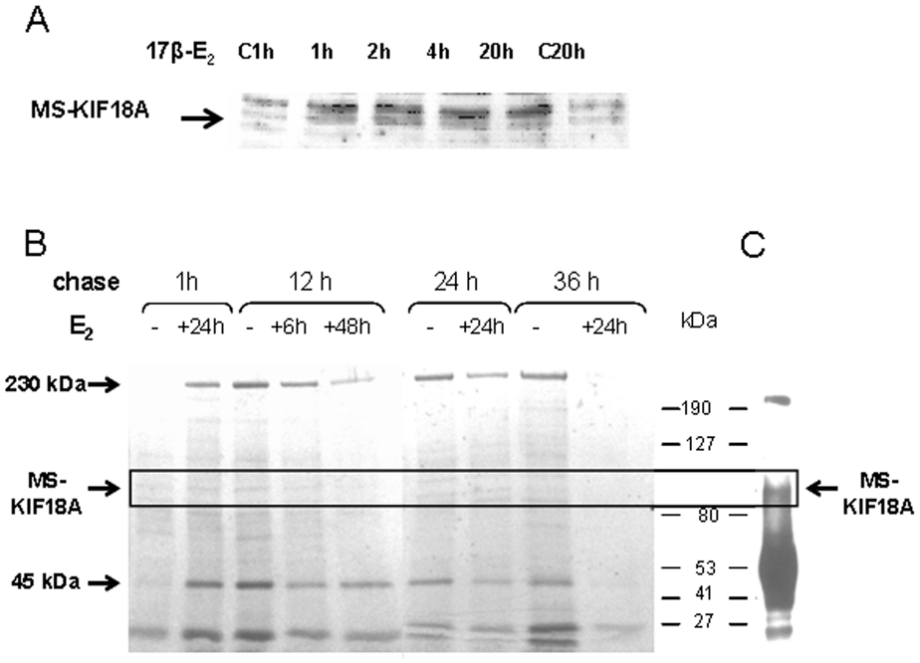
Estrogen effects on MS-KIF18A protein expression and turnover. (A) Lysates of MBA-15 cells challenged with 10^−8^ M 17βE_2_ for 1 h, 2 h, 4 h and 20 h were analyzed by WB with anti-MS-KIF18A and compared to the untreated control at 1 h (C 1 h) and 20 h (C 20 h). (B) MBA-15 cells were pretreated with 10^−8^ M 17βE_2_ for duration of 6 h, 24 h or 48 h, metabolic labeled with Met/Cis-S^35^, chased for various time periods from 1 h to 36 h, lysed, IPed with anti-MS-KIF18A and loaded on SDS-PAGE gel. (C) A 100 kDa protein confirmed as MS-KIF18A by IP and WB with anti-MS-KIF18A. Results are of representative experiment of a series repeated three times.

Additionally, we followed the MS-KIF18A protein in MBA-15 cells treated by 17βE_2_ for defined periods from 1 h to 20 h. The cell lysates were separated on SDS-PAGE gel and analyzed by WB with anti-MS-KIF18A. We noted an increase in MS-KIF18A protein expression already after 1 h of treatment that was maintained until 20 h (Figure 9A). In addition, MS-KIF18A protein turnover was analyzed in presence or absence of estrogen applying metabolic labeling with Met/Cis-S^35^ on MBA-15 cells. The cells were pretreated for 6 h, 24 h or 48 h with 17βE_2_, radio-labeled for 1 h and then chased at 4 time points from 1 h to 36 h. At each time point cell lysates were IPed with anti-MS-KIF18A, separated on SDS-PAGE and exposed to developing film (Figure 9B). A 100 kDa band was identified and confirmed by IP and WB analysis as MS-KIF18A (Figure 9C). We have shown MS-KIF18A protein degradation in period of 24 h to 36 h after cells' labeling. The estrogen treatment induced an increase of MS-KIF18A synthesis (1 h chase), accelerated protein turnover (12 h chases) and shortened its half-life (36 h chase). In addition, other prominent proteins of 230 kDa and 45 kDa (earlier identified as actin [11]) were Co-IPed by anti-MS-KIF18A (Figure 9B).

In summary, we demonstrated the association between MS-KIF18A and ERα recombinant and endogenous proteins analyzed in MBA-15 cells. These proteins' association was regulated by 17βE_2_ and the complex appeared more prominent in cytoplasm. At the molecular level, we demonstrated estrogen dependent activation of MS-KIF18A promoter measured by luciferase assay and an estrogenic increase in ERα and pcJun binding to the promoter. The regulation of MS-KIF18A mRNA and protein expression by estrogen was demonstrated *in vivo* in mice and rats as well as in cell systems.

## Discussion

MS-KIF18A is a member of kinesin family, which functions as motor protein that binds to microtubule and uses the energy derived from ATP hydrolysis to move along the cytoskeleton. Previous studies analyzed the MS-KIF18A using bioinformatics and biochemical tools suggested the estrogen receptor alpha (ERα) as a putative cargo [10]–[12]. The present study elaborated on two aspects; one highlighted on the interactions between MS-KIF18A and ERα and the second studied the expression of MS-KIF18A under the paradigm of estrogen which applied *in vivo* and *in vitro* models.

The complex formation between motor proteins and their cargo comes from the motifs that play a role in protein-protein interactions. Bioinformatics analysis of MS-KIF18A protein sequence revealed several motifs of interaction with nuclear receptors (NR-boxes and ΦXXΦΦ-like motifs) and a region homologous to ERα in the coiled coil and the cargo-binding domains of the kinesin. Co-transfection of MS-KIF18A and ERα in COS-7 null cells and also Co-IP of endogenous proteins in MBA-15 cells demonstrated such association [10], [12]. Here, we elaborated on the nature of these proteins interactions in *in vitro* assay using three constructs of recombinant MS-KIF18A and ERα. We have shown that full length MS-KIF18A or its truncated forms bind the ERα. Thus, we propose a putative function for the NR motifs localized at the coiled-coil region and cargo-binding domain of the MS-KIF18A, in mediating the proteins interactions.

Estrogen is recognized to activate numerous of transcriptional events in many cell types. Estrogen receptor localization was noted at various cell compartments that are down regulated in cells treated by 17βE2 [43], [44]. ERα shuttles between different cellular compartments including cell membrane, cytoplasm and nucleus [12], [30], [43]. A small portion of ERα is localized at the caveolar fractions of the plasma membrane [12], [45], [46], however the receptor is mostly localizes in the nucleus [21], [42], [47]. A connection between ERα mobility and a putative cytoskeleton protein was suggested, but no candidate protein has been identified thus far [43]. Herein, we have shown that fractionated cells analyzed by WB localizes the ERα is mainly at the nucleus/membrane compartments, while MS-KIF18A is prevalent in cytoplasm [11]. The association between MS-KIF18A and ERα is higher in the cytoplasm, confirming the kinesins' action in this compartment. We have also shown that the complex formed between these proteins is stimulated by 17βE_2_.

ERα signaling involves activation of MAPKs such as p38 and ERK1/2 [31], resulting with various cell response. pERK protein was earlier detected in association with ERα and MS-KIF18A, suggesting a role for the MS-KIF18A in non-genomic activation of ERα through the MAPK pathway [12]. Herein, we elaborated on the protein complex between MS-KIF18A and ERα revealing it regulation by estrogen. It is recognized that ERα binds NF-κB in various cells affecting cellular process such as inhibition of inflammatory or during cell apoptosis [35]. NF-κB transcription factor dimerize the p65 and p50 subunits to hetero-complex [48]. We detected an association between both NF-κB subunits and ERα, while MS-KIF18A binds only the p50 subunit.

Estrogen regulates numerous cellular functions including the remodeling of the cytoskeleton proteins and their composition. The cytoskeleton is a dynamic network of proteins that undergoes restructuring during cell division, formation of cell-cell or cell-ECM interactions and cell migration. Cytoskeleton plays a role in controlling of cells shape and influences gene expression [22], [49], [50]. We earlier reported that 17βE_2_ affects the composition of cytoskeleton proteins, such as thropomyosin and tubulin and reorganization of actin fibers in MBA-15 cells [22]. Estrogen induces the remodeling of both the F-actin and the intermediate filament [50]. It was shown also in vivo, in OVX-estrogen depleted rats an up-regulation of tropomyosin 2β and tropomyosin 1α expression by 17βE_2_ [51]. Earlier we have shown the association between MS-KIF18A and cytoskeleton proteins tubulin and actin [11]. The current study provides new insights on the impact of estrogen on the complex formed between ERα and MS-KIF18A.

The expression of MS-KIF18A mRNA and its' regulation by estrogen was analyzed *in vivo* in rats and mice. We have noticed an age difference in MS-KIF18A expression: mRNA was higher expressed in young animals then in old ones. Such differences may account for the decrease in estrogen levels with aging [17], [52], [53] suggesting a hormonal role in regulation of MS-KIF18A expression *in vivo*. This observation was strengthened using OVX-estrogen depleted rats which measured a decline in MS-KIF18A message as compared to sham rats. When the OVX-rats were treated with 17βE_2_ we have shown the restoration of MS-KIF18A mRNA levels.

To unravel the mechanism of estrogen effects on MS-KIF18A expression we analyzed the kinesin promoter activity by luciferase reporter gene and ChIP assays. It is known that ERα activates promoters when binds directly to palindrome ERE or half-site ERE [39], [54], [55] or indirectly via accessory proteins (such as Fos and Jun) at AP-1 binding sites or to GC-rich sequences via complex with Sp1 [40], [56]. ERα also stimulates gene expression via interaction with nuclear receptor NF-κB and this complex binding to promoters of target genes [48]. Herein, we used the cloned promoter of MS-KIF18A that contains non-palindromic half-site ERE, AP-1 and NF-κB binding sites. The MS-KIF18A promoter was cloned under luciferase reporter and transfected to MCF-7 cells. When these cells were treated with either 17βE_2_ or ICI-182,780 we have noted a similar inducible effect of the promoter activity while the combined 17βE_2_/ICI-182,780 treatment had an additive effect. MS-KIF18A mRNA levels were quantified by qPCR and have shown an increase in cells treated with either 17βE_2_ or ICI-182,780. The rational for the ICI-182,780 effect shown here lies in the fact that this drug is no more considered as an estrogen antagonist and reports present a role for its agonistic action. The agonist property of ICI-182,780 was observed *in vivo* on bone growth [57]. Cells derived from human breast tumor tissues were analyzed on HTS affymetrix gene chip resulted with an up-regulation of gene expression when treated by estrogen and even more by ICI-182,780 [58]. Modulation by ICI-182,780 resulted with up regulation of quinine reductase in MCF-7 cells [59], ERRα in SKBR3 cells [60] and spinophilin in hippocampus neurons [61]. The promoter activation by ICI-182,780 is proposed to act via ER binding on AP-1 sites, but not via ERE [62]. Moreover, the ERα-ICI-182,780 complexes that sequester transcriptional repressors away from AP-1 sites allow an unrestricted transcription [63], [64]. From the current study the indication is that binding of 17βE_2_ and ICI-182,780 to ERα increases the receptor binding to MS-KIF18A promoter and allows its activation, leading to an increase in message transcription. Using the ChIP analysis we have shown that ERα and pcJun bind to MS-KIF18A promoter in MCF-7 and MBA-15 cells that are regulated by 17βE_2_ and by ICI-182,780. In another study, we have shown that ERα and cJun are differentially regulated when bind to SVEP1 promoter. In cells treated by 17βE_2_ it was shown an increase of the TF binding but not when treated by ICI-182,780 [65].

Complementary with reporter gene and ChIP assays we analyzed the role of estrogen on MS-KIF18A mRNA and protein expression. It was noted that MS-KIF18A mRNA expression increased after cells were challenged with 17βE_2_ or ICI-182,780. Furthermore, inhibition of transcription with ActD and 17βE_2_ stabilized MS-KIF18A mRNA and decreased its' degradation. MS-KIF18A protein levels also increased following 17βE_2_ treatment. Metabolic labeling assay confirmed that 17βE_2_ accelerates MS-KIF18A turnover. Taken together, the results elaborated on the effect of estrogen on MS-KIF18A expression, lifetime and degradation.

In summary, we have shown the interaction between MS-KIF18A and ERα as its cargo in *in vitro* and *in vivo* assays using different biochemical and molecular approaches. Moreover, the involvement of kinesin in ERα signaling was demonstrated. Furthermore, we have shown for the first time regulation of MS-KIF18A mRNA expression and protein turnover by estrogen, implying the kinesins' function in estrogen-dependent manner. These results can lead to further investigation of metabolic regulation in the mesenchymal stem cells that play a role in estrogen regulated maintenance of bone in metabolic diseases or in cancer.

## Materials and Methods

### Animals and Experimental Design

ICR mice age 4-month (young) and 12-month (old) old (n = 10 in each group). Fischer 344 male and female rats age 90-day (young) and 11-month (old) old (n = 13–18 rats in each group from both sex). Female rats were subdivided into a control sham group (abdominal midline incision), ovariectomized (OVX) rats or OVX rats that were implanted with 90-day slow-release pellets of 17β-E_2_ [17]. Animals were maintained and treated according to the Institutional Animal Care and Use Committee at the Tel Aviv University.

### Cell culture

We used two cell lines known for their response to estrogen: MBA-15, a pre-osteogenic stromal cell line [22] and MCF-7 breast carcinoma cell line. Both were cultured in growth medium; Dulbecco's Modified Essential Medium (DMEM) (Gibco, USA) with the addition of 10% heat-inactivated fetal calf serum (FCS) (Biological Industries, Israel), supplemented with 1% glutamine and 1% penicillin/streptomycin in a humidified atmosphere of 5% CO_2_ at 37°C. Before cells' were treatment with 10^−8^ M 17βE_2_ or 10^−7^ M ICI-182,780, they were incubated in 3% serum stripped medium for 48 h. Where specified, cells were treated with 5 µg/ml actinomycin D (Sigma, USA) a transcription inhibitor.

### Bioinformatics analysis

60 kb of genomic sequence at 5′-flanking upstream the transcription start site (TSS) of MS-KIF18A gene was analyzed to identify the gene-putative promoter. The analysis applied Promoter 2.0 Prediction Server (http://www.cbs.dtu.dk/services/Promoter) for promoter definition and MatInspector software (http://www.genomatix.de) for transcription factor binding sites identification. All primers were constricted using Primer3 Software (http://frodo.wi.mit.edu/cgi-bin/primer3).

### Putative MS-KIF18A promoter cloning and activity

1.5-kb 5′-flanking upstream the transcription start site (TSS) promoter sequence segment was amplified from genomic DNA using the 5′ TACCAAGACCAGCAGCACAC and 3′ TAAGGAGATCCCTGCCCTTC primers. The PCR fragment was verified by sequencing then restricted by Bgl II and Bcl I and a 1.3-kb segment was cloned upstream of a luciferase reporter gene into pGLuc-basic vector (New England BioLabs, USA). The MS-KIF18A promoter reporter plasmids named pGLuc-K.

MCF-7 cells seeded in 6-well plates, after 48 h cells were transfected with 1.3 µg of the pGLuc-K or promoterless pGL3-basic along with 0.3 µg of β-galactosidase expression plasmid (pCMVβ; Clontech, Palo Alto, CA), using the jetPEI™ transfection reagent (Polyplus Transfection, Illkirch, France). After 24 hrs media was changed to 3% serum stripped medium supplemented with 10^−8^ M 17β-E_2_ and 10^−7^ M ICI-182,780 for 1 h or 24 h then medium was collected and cells were harvested for luciferase activity (New England BioLabs, USA) and β-galactosidase activity [66]. Promoter activities are expressed as luciferase values normalized to β-galactosidase levels.

### mRNA and gene expression analysis by real-time quantitative PCR

Total RNA was extracted from cells (EZ RNA kit, Biological Industries, Beit Haemek, Israel) and reverse transcribed to cDNA using Reverse-iT 1^st^ Strand Synthesis Kit (ABgene House, AB-0789) and oligo-dT primer (Takara Shuzo Co. Ltd., Seta, Japan). The cDNA used as template for polymerase chain reaction (PCR), using primers for MS-KIF18A from human, rat and mouse (Table 1). Amplified PCR products were detected by SYBR Green (ABgene House, USA). Verification of a single product amplified was checked for each primer pair by analysis of product melt curves on (MxPro™ QPCR Software, Stratagene, USA). MS-KIF18A cDNA PCR products were subjected to dissociation curve analysis resulting with fluorescence peak corresponding to the MS-KIF18A product centered at 80°C in rats and at 82°C in mice and humans. Primer-dimmers were distinguishable at 76°C. Level of expression for PCR products was normalized to G3PDH gene expression. Experiments were performed with triplicates for each data point.

**Table 1:** Primers used for mRNA expression analyzed by PCR.

Gene	Sequence	
MS-KIF18A Mouse cDNA	sense	5′ TCAATCAAAATGTCCGTAT 3′
	antisense	5′ GGCTTTCTGTTCTTCATAGG 3′
MS-KIF18A Human cDNA	sense	5′ GTGCCATCCTACATGGCAATG 3′
	antisense	5′ TGTCGAACACGTTTGGCAAA 3′
MS-KIF18A Rat cDNA	sense	5′ CAAAATGGTGATATTCCCGAGG 3′
	antisense	5′ CAGCCAGAGTCATCATGTGTCC 3′
G3PDH	sense	5′ ACCACAGTCCATGCCATCAC 3′
	antisense	5′ TCCACCACCCTGTTGCTGTA 3′

### Chromatin immunoprecipitation (ChIP) analysis of regulatory factor binding to putative MS-KIF18A promoter in human and mouse cells [67]

For assay, DNA was extracted from input chromatin fractions and complex was immunopercipitated with anti-ERα (Stressgen, Canada) or with anti-pcJun (Santa Cruz Biotechnology, USA). DNA served a template to amplify promoter of MS-KIF18A gene, with specific primers (Table 2) using real-time quantitative PCR. Input DNA was used as a positive control and for standard curve. The fluorescence peak dissociation corresponding to PCR product centered at 85°C and was distinguishable from the peak of primer-dimmer centered around 79–80°C.

**Table 2:** Primers used for ChIP analysis.

Gene	Sequence	
MS-KIF18A Mouse promoter	sense	5′ TTTTACAGGCCCGCAGACTC 3′
	antisense	5′ GAAGCAGCCACCTGGGATATT 3′
MS-KIF18A Human promoter	sense	5′ ACGTGATGACATCACGCGAG 3′
	antisense	5′ CTTTAATGTCCGCCTCCCAG 3′

### Immunological methods

Cells' were collected for immunoprecipitation (IP) or whole lysate (WL) analysis; SDS-PAGE gel and Western blot (WB). Cells were washed twice with ice-cold PBS and collected in presence of protease inhibitors (phenylmethylsulfonyl fluoride, PMSF, 1 mM; 1-chloro-3-tosylamido-4-phenyl-2-butanone, TPCK, 10 µg/ml; aprotinin, 10 µg/ml and phosphatase inhibitors cocktails I and II (Sigma, USA). Samples were spin down at 1500 rpm for 4 min, lysed in lyses buffer consisting of 50 mM Tris pH 7.5, 150 mM NaCl, 1% NP-40; protease and phosphatase inhibitors; incubated for 20 min at 4°C and centrifuged at 16,000×*g* for 5 min. For WL the samples were resuspended in Lamelli sample buffer and boiled for 3 min. For IP the 1 µl of the antibody of interest and 25 µl Protein-A sepharose beads (RepliGen, USA) were added to lysates and samples were incubated overnight at 4°C. Immunocomplexes were precipitated at 16,000×*g* for 1 min and washed four times with lyses buffer. The washed beads were resuspended in Lamelli sample buffer and boiled for 3 min. The proteins were separated on 8% SDS-PAGE for 2 h 30 min and transferred to nitrocellulose for 1 h 30 min. For Western blot, the membranes were blocked with 5% BSA in TBST (50 mM Tris, pH 7.5, 150 mM NaCl, 0.1% Tween-20, Sigma, USA) for 1 h at RT or overnight at 4°C and then incubated with primary antibody. The membranes were washed with TBST and incubated with goat anti-rabbit or goat anti-mouse conjugated to biotin (Dako, Denmark) for 40 min at RT and with extravidin-peroxidase for 30 min at RT (Sigma, USA) for detection with chemiluminescent substrate (Pierce, USA).

### Antibodies

Polyclonal anti-MS-KIF18A (1∶1300) [10], [12]; monoclonal anti-MS-KIF18A (1∶500) [11]; anti-ERα (1∶800) (SRA-1010, Stressgen, Canada), anti-p65 and anti-p50 (Santa Cruz, USA).

### Metabolic Labeling and Immunoprecipitation

MBA-15 cells were grown to 70% confluence in 100 mm dishes, cells were pretreated with 10^−8^ M 17βE_2_ for 6 h, 24 h and 48 h. The medium was replaced with medium depleted of serum for 1 h and cells were metabolically labeled in the presence or absence of 17βE_2_ for 1 h at 37°C using Redivue promix S^35^ label (200 µCi/sample; Amersham) in methionine and cysteine-free Dulbecco's modified Eagle's medium (DMEM) supplemented with 3% stripped FCS. At the end of each time point, cells were washed twice with PBS and with DMEM supplemented with 3% stripped FCS with or without 17βE_2_ for variable periods of time. Cells were lysed in 50 mM Tris, pH 7.5, 150 mM NaCl, 1% NP40 and protease and phosphatase inhibitors and MS-KIF18A was immunoprecipitated overnight at 4°C using anti-MS-KIF18A antibody and protein A sepharose beads (RepliGen, USA). The immunocomplex was washed three time with lysis buffer, resolved in 8% SDS-PAGE, and detected using Kodak BioMax MS Film.

### Cell fractionation

Cells (1×10^7^) were washed twice with 3 ml of cold PBS, resuspended in 100 µl Buffer A (10 mM HEPES, pH 7.4, 10 mM KCl, 1.5 mM MgCl_2_, 0.5 mM DTT, 0.025% NP-40) with protease inhibitors (1 µg/ml aprotinin, 1 µg/ml TPCK, 1 µg/ml pepstatin A, 0.2 mM PMSF) and incubated on ice for 20 min followed with centrifugation at 7500 rpm for 10 min at 4°C and the cytoplasm extract was removed, frozen and stored at −80°C. The pellet was resuspended in 50 µl Buffer B (20 mM HEPES, pH 7.4, 420 mM NaCl, 1.5 mM MgCl_2_, 0.5 mM DTT, 0.2 mM EDTA, 25% Glycerol) with protease inhibitors. The nuclear suspension was stirred vigorously on ice for 30 min. The sample was centrifuged at 15 000 rpm for 12 min at 4°C, and the nuclear/membrane extract was frozen and stored at −80°C. The protein concentration of cytoplasm and nuclear/membrane extracts was determined by the NanoDrop (ND-1000 Spectrophotometer, NanoDrop Technologies, Inc., USA).

### Binding assay for recombinant proteins

MS-KIF18A recombinant proteins were expressed at the Structural Proteomics Center, Weizmann Institute of Science, Israel. Recombinant ERα was purchased (Sigma, USA). For experiments we used 0.25 µg of protein and pull down was performed in presence of either poly or monoclonal anti-MS-KIF18A, anti-ERα with 25 µl Protein-A sepharose beads (RepliGen, USA) overnight at 4°C. Then beads were spanned down, re-suspended with loading buffer and protein complex was analyzed by western blot.

### Statistical analysis

Statistical analyses were carried out by Student's *t*-test, where values of p<0.05 are statistically significant. * is p-value≤0.05, ** is p-value≤0.01, *** is p-value≤0.001.
